# MicroRNAs: A Novel Approach for Monitoring Treatment Response in Major Depressive Disorder?

**DOI:** 10.3390/ncrna11020021

**Published:** 2025-03-03

**Authors:** Cristina-Sorina Cătană, Monica Mihaela Marta, Daniel Ungureanu, Cătălina-Angela Crișan

**Affiliations:** 1 Department of Medical Biochemistry, “Iuliu Hațieganu” University of Medicine and Pharmacy, 8 Victor Babeș Street, 400012 Cluj-Napoca, Romania; ccatana@umfcluj.ro; 2 Department of Medical Education, “Iuliu Hațieganu” University of Medicine and Pharmacy, 8 Victor Babeș Street, 400012 Cluj-Napoca, Romania; mmarta@umfcluj.ro; 3 Department of Pharmaceutical Chemistry, “Iuliu Hațieganu” University of Medicine and Pharmacy, 8 Victor Babeș Street, 400012 Cluj-Napoca, Romania; 4 “Prof. Dr. Ion Chiricuță” Institute of Oncology, 34–36 Republicii Street, 400015 Cluj-Napoca, Romania; 5 Department of Psychiatry and Pediatric Psychiatry, “Iuliu Hațieganu” University of Medicine and Pharmacy, 8 Victor Babeș Street, 400012 Cluj-Napoca, Romania; ccrisan@umfcluj.ro; 6 First Psychiatric Clinic, Cluj County Emergency Hospital, 43 Victor Babeș Street, 400012 Cluj-Napoca, Romania

**Keywords:** microRNA, major depressive disorder, biomarker, antidepressant, miRNA therapeutics, extracellular vesicles

## Abstract

Major depressive disorder (MDD) is one of the most prevalent psychiatric disorders, with an increasing incidence each year and an important socioeconomic burden. Although new treatments are continuously being developed, there is no effective monitoring method to determine the suitability of treatment and ensure positive outcomes. Therefore, patients often struggle with ineffective antidepressants and their potential adverse effects, which halts any future progress in managing the disorder. Considering the potential of microRNAs (miRNAs) as biomarkers for various pathologies and the increasing evidence of the modulation of several genes involved in MDD, this minireview aimed to evaluate the literature data on the impact of miRNAs in MDD and their usefulness in monitoring treatment response. The correlations between antidepressants and the expression of several miRNAs support the existence of a common epigenetic mechanism of antidepressants and explain the epigenetic differences influencing treatment efficacy in MDD.

## 1. Introduction

Major depressive disorder (MDD) is considered a serious threat to global health and one of the causes of disability affecting working life, relationships, and physical health. It is estimated that approximately 5% of people worldwide suffer from depression, with a peak period of onset in the mid-20s, thus affecting working-age individuals and causing negative socioeconomic consequences [1].

Untreated MDD can lead to an increased rate of suicide among sufferers, consequently affecting their caregivers/families and subsequently increasing the socioeconomic burden [2]. Currently, there are various treatment options available for MDD, including different forms of psychotherapy and a wide range of antidepressants. However, about 30% of MDD patients are prone to becoming treatment-resistant, i.e., having an inadequate response to at least two antidepressants despite adherence to adequate treatment, although a significant percentage of treatment-resistant MDD patients are in fact pseudo-resistant due to inadequate treatment and non-adherence [3]. Besides psychometric scales and subjective information from patients, currently, there is no certified biomarker for evaluating the efficacy of antidepressants. Therefore, it is difficult to establish an effective antidepressant after the first attempt, so patients may try several other antidepressants and experience possible adverse effects. This could further lead to treatment non-adherence and distrust in MDD treatment options.

In recent years, there has been increasing evidence regarding the potential use of microRNAs (miRNAs) and other non-coding RNAs (ncRNAs) in tracking the treatment response in MDD. Evidence has been observed either for antidepressants or electroconvulsive therapy (ECT).

Previous research has demonstrated the crucial role of short RNA molecules as major components of cellular gene modulation and function in the etiology of depression. RNA molecules can control and modify the post-transcriptional expression level of target miRNAs. Moreover, extracellular vesicles (EVs) deliver a set of regulatory miRNAs, thus exchanging molecular signals between the blood and the central nervous system (CNS). This is significant since it has already been shown that certain peripherally identified miRNAs are dysregulated in different pathological phenotypes seen in depression [4]. Our minireview aimed to evaluate the growing body of evidence for the use of miRNAs as a target for RNA-based therapies, as well as some of the available literature data regarding therapy monitoring and treatment response in MDD using miRNAs.

## 2. MicroRNAs in Major Depressive Disorder

MiRNAs have been considered important regulators of several processes in the CNS. Hence, it has been hypothesized that miRNA modulation can play a significant role in several brain disorders, including psychiatric disorders [5]. The therapeutic role of miRNAs in MDD has been extensively studied over the last decade, and the possibility of employing miRNAs as future therapy in MDD is becoming increasingly feasible [4,6,7,8,9,10].

Referring strictly to depression, notable miRNAs with clear therapeutic implications or serving as feasible targets include miR-30a, miR-133b, miR-26a-2, let-7, miR-144, miR-9, miR-16, with current studies focusing on the potential modulation of the let-7 family for therapeutic purposes. Lentiviral technologies have the advantage of being used as non-invasive biomarkers, with documented applications in the exploration, monitoring, and early detection of Alzheimer’s disease (AD). They have also been used to examine the effects of physical activity on its epigenetic regulation. Of particular importance is let-7d, known for its promising anxiolytic and antidepressant effects [11,12,13,14].

The review by Luoni and Riva mentioned several dysregulated miRNAs associated with MDD. For example, miR-30e ss178011483, a T risk allele, induced a longer P300 waveform latency in MDD patients with the C/T genotype compared to the C/C genotype, which was clinically associated with a decrease in cognitive functioning. Similarly, miR-182 rs76481776, another T risk allele, increased the expression of miR-182 in MDD patients, which downregulated ADCY6, CLOCK, and DSIP genes, clinically resulting in a dysfunctional circadian clock period [5].

The rs17228616 AChE allele weakens the interaction between acetylcholine (AChE) and miR-608 in MDD patients. This results in an overexpression of miR-608, which can suppress its other targets, clinically manifesting as an increased risk of anxiety and elevated blood pressure [5].

Another signature feature of miRNAs observed in MDD was their overall downregulation in the prefrontal cortex (PFC), as concluded by a study on depressed suicide subjects [5].

Several other miRNAs have been associated with MDD through the dysregulation of inflammatory genes (miR-155), glucocorticoid receptor (GR) activity decrease (miRNA-155), and reduction of the brain-derived neurotrophic factor (BDNF) by overexpression of miR-30-5p, miR-124a, miR-16, and miR-182 [5].

MiRNAs have also been associated with treatment-resistant depression (TRD). According to the literature, TRD is characterized by increased activation of the inflammasome system, hyperactivity of the hypothalamic–pituitary–adrenal axis, and impaired sensitivity of the GR [15]. Minelli et al. conducted a study involving the role of proteasome system dysregulations in treatment resistance mechanisms in MDD. Given the role of the ubiquitin–proteasome system in neuropsychiatric disorders, they examined the expression of the PSMA7 (proteasome alpha type 7 subunit), PSMD9 (26S proteasome non-ATPase subunit 9), and PSMD13 genes. The results showed that subjects with the homozygous GG genotype of the PSMD13 rs3817629 allele had double the risk of developing a treatment-resistant form of MDD [15].

Additionally, TRD in MDD patients was associated with increased inflammasome activation and glucocorticoid resistance. The excessive inflammasome activation was attributed to the increased expression of purinergic receptor P2RX7 and pro-inflammatory cytokines/chemokines/miRNAs, while glucocorticoid resistance was induced by a decreased expression of the GR miRNA and an increased expression of FK506 binding protein 5 (FKBP5) miRNA [16]. According to a study conducted by Cattaneo et al., the expression of the following miRNAs distinguished treatment-resistant patients from responsive patients with depression: P2RX7, IL-1-β, IL-6, TNF-α, CXCL12, and GR [16]. Other miRNAs that could potentially be used as biomarkers for diagnosis and treatment monitoring in MDD were summarized in the systematic review of Carneiro et al. The proposed miRNAs were miR-1202, miR-135, miR-124, and miR-16. Each of them is involved in the expression of BDNF [17].

Given their essential antidepressant properties demonstrated in animal models, certain miRNAs could be candidates for miRNA replacement therapy, which reinstates cellular programs active in physiological conditions. Examples of miRNAs with a significant impact in MDD include miR-26a-2, which acts on the serotonergic neurons by targeting the serotonin receptor 1A gene (HTR1A) [4]. The knockdown of this receptor increased anxious behavior. Another example is miR-101, which reversed specific depressive behaviors by modulating them in rats and targeting dual-specificity phosphatase 1 (DUSP1) [18]. Moreover, combining miR-135a with traditional antidepressants, which upregulate its expression, led to a decline in inflammatory markers in certain experimental models [18,19,20,21,22,23].

A noteworthy experimental study revealed the importance of miR-139-5p in depression. The chronic unpredictable mild stress (CUMS)-induced mouse model was used to observe a decrease in miR-139-5p levels, which was correlated with depression-like clinical symptoms. Additionally, using the adeno-associated virus (AAV) technology, the increased hippocampal level of miR-139-5p expression activated the cAMP/PKA signaling pathway, which induced antidepressant-like effects in mice [24].

Similarly, it was demonstrated that the increase in miR-144 levels led to observed antidepressant effects in the CUMS rat model. This miRNA is of particular importance since it proved to be reduced in MDD patients and was overexpressed in experimental models using mood stabilizer treatments. The same animal model was used to highlight the crucial role of miR-124-3p, upregulated in the hippocampus, in decreasing proinflammatory cytokines in microglia. Recent research on the alleviation of neurodegenerative processes and the improvement of cognitive functions identified the presence of miR-124-3p within microglial EV miRNA cargos, with miR-124-3p acting as an efficient therapeutic intervention in depression [25,26,27,28,29].

Regulation of cargo selection in exosome biogenesis is dictated by a complex interplay involving the syntenin–Alix pathway, tetraspanins, lipids, the endosomal sorting complex required for transport (ESCRT), and arrestin domain-containing protein 1. Once secreted, EVs act as novel mediators of extracellular matrix degradation by navigating through vascular barriers such as the blood–brain barrier (BBB) in both directions, entering the systemic circulation, and preparing a permissive environment for various neurodegenerative conditions [30,31,32,33,34,35].

Due to their nanoscale dimensions (30–200 nm), exosomes can cross the BBB. While the exact mechanisms of this means of transport are still under investigation, transcytosis appears to be a plausible route. Upon reaching the target site, the cargo is released following internalization by the recipient cell through endocytosis and then transferred to lysosomes after fusion with the endosomal or lysosomal membrane in an acidification-dependent manner (pH < 6.0) [35,36,37].

## 3. MicroRNAs in Monitoring Treatment Response

The recent literature on antidepressants highlights an epigenetic mechanism of antidepressants, which could help predict their efficacy and explain some of their adverse effects not previously related to their pharmacological mechanism. For example, a study conducted by Lin et al. showed an increase in the expression of miR-183 and miR-212 after a four-week treatment with either selective serotonin reuptake inhibitors (SSRIs) or serotonin and noradrenaline reuptake inhibitors (SNRIs) [38]. Similarly, Hung et al. showed that the expression of exosomal miRNAs miR-21-5p and miR-155 was lower in patients who achieved remission after antidepressant treatment [39].

### 3.1. Tricyclic Antidepressants

According to Issler et al. (as cited by Luoni and Riva), miR-135a, which is suppressed in serotonergic neurons in the raphe nucleus of MDD patients, was strongly associated with the serotonin transporter (SERT) and serotonin receptor-1A (5HT-1A). Chronic treatment with imipramine was able to upregulate miR-135a, although the overexpression of this miRNA maintained the depression-like phenotype because it downregulated the SERT and the 5HT-1A autoreceptor [5,22]. Additionally, a study by Grosse et al. (as cited by Goossens et al.) showed that imipramine was capable of increasing the number of regulatory T cells (Treg) and helper T cells (Th) Th2, which were present in lower proportions in MDD patients [40,41].

The systematic review by Goossens et al. also included a study conducted by Weizman et al. demonstrating that a 4-week treatment with clomipramine normalized the production of inflammatory cytokines interleukin-1β (IL-1β), IL-2, and IL-3-like activity (IL-3LA), which are decreased in MDD patients. This is an important finding since inflammatory dysregulations have long been associated with the etiopathology of MDD [40,42].

According to Alcocer-Gόmez et al. (as cited by Goossens et al.), amitriptyline was capable of reversing the increase in miRNAs that encode caspase-1 and NLR family pyrin domain containing 3 (NLRP3), which are overexpressed in MDD [40,43].

### 3.2. Serotonin Selective Reuptake Inhibitor Antidepressants

#### 3.2.1. Fluoxetine

SSRIs are by far the most studied antidepressants regarding their possible epigenetic mechanisms. According to Issler et al. (as cited in the review by Luoni and Riva), chronic treatment with fluoxetine was also capable of upregulating the mir-135a level, similarly to imipramine [5,22]. The affinity of fluoxetine for the serotonergic neurons in the raphe nucleus was also demonstrated by the animal model study by Baudry et al. included in the same review. They proved that fluoxetine selectively upregulated miR-16, which targets SERT expression. Fluoxetine presents different epigenetic mechanisms depending on the length of administration [5,44]. An animal model study by Lo Iacono et al. showed different alterations in the 5HT-2C receptor in the dorsal raphe nucleus, expressed by miR-34a, following acute and chronic fluoxetine treatment. Chronic fluoxetine suppressed miR-34a expression, which upregulated the expression of 5HT-2C, which was clinically translated into behavioral effects, specifically the impairment of an adequate response to an acute threat [45].

Another epigenetic mechanism of fluoxetine was reported by Tao et al., who observed that fluoxetine inhibited the expression of miR-135b-5p and regulated the expression of silent information regulator 1 (SIRT1). The role of SIRT1 in the etiopathology of MDD is constantly expanding, as it was observed that MDD patients have reduced levels of SIRT1, and its dysregulations are responsible for depression-like behaviors. Additionally, by inhibiting the expression of the same miRNA, fluoxetine was able to reduce the depression-like behavior induced by the CUMS, observed in the decreased inflammatory factors IL-1β, IL-6, and TNF-α in hippocampal mouse samples [46].

#### 3.2.2. Fluvoxamine

When fluvoxamine was used as an add-on to antipsychotic treatment in schizophrenia patients, there was a 40% decrease in the amount of protein kinase C beta (PKCβ)-2 miRNA, according to Silver et al. (as cited in the review by Goossens et al.). However, the decrease could not be attributed to fluvoxamine or antipsychotic treatment since no control group was studied [40,47].

#### 3.2.3. Sertraline

A study conducted by Ahmadimanesh et al. showed that a 100-day treatment with sertraline increased the expression of miR-16 and decreased the expression of miR-124 and miR-132. While the increase in miR-16 is important since it regulates SERT expression, both miR-124 and miR-132 modulate the expression of the BDNF gene, and their reduction increases the levels of BDNF [48].

#### 3.2.4. Paroxetine

Paroxetine has effectively demonstrated an increase in miR-451a expression and a decrease in miR-34a-5p and miR-221-3p expression following an 8-week treatment in a study conducted by Kuang et al. While their exact role has not been fully deciphered, the authors concluded that these miRNAs could be considered potential predictors of the efficacy of antidepressant treatment, based on how their levels were modified during paroxetine treatment according to the Hamilton Depression Scale (HAMD) scores [49].

#### 3.2.5. Citalopram

Citalopram and escitalopram are probably the SSRIs with the most studied epigenetic mechanisms. A study conducted by Lopez et al. demonstrated that 8 weeks of citalopram treatment normalized miR-1202 levels in MDD patients. This miRNA is particularly important because it remains at lower levels in patients who are resistant to at least one antidepressant. Thus, the authors postulated that the peripheral expression of this miRNA at baseline may be predictive of treatment response to antidepressants [50]. According to Ahmadimanesh et al., 100-day treatment with citalopram increased the expression of miR-16, but did not decrease the expressions of miR-124 and miR-132 compared to sertraline [48]. Therefore, both these studies found epigenetic differences between sertraline and citalopram, but only sertraline increased BDNF levels. However, an earlier study conducted by Fang et al. showed that a 2-month citalopram treatment was able to decrease miR-132 levels, but not miR-124 levels, as there were no differences between the treatment-free and citalopram groups of MDD patients [51]. Citalopram was also analyzed in a study conducted by Wang et al., demonstrating that it could downregulate miR-155 and, thus, upregulate the expression of SIRT1 [52].

#### 3.2.6. Escitalopram

In an extensive study conducted by Bocchio-Chiavetto et al., the expression of 30 different miRNAs from peripheral blood was evaluated following a 12-week treatment with escitalopram. The results showed that 28 miRNAs were upregulated and 2 were downregulated, thus affecting several pathways involved in neuronal brain function [53]. The same treatment regimen was applied in a study by Cattaneo et al., who observed that escitalopram increased BDNF mRNA levels [54].

A study conducted by Enatescu et al. using the same 12-week escitalopram treatment regimen identified five miRNAs commonly regarded as dysregulated in MDD: miR-146a-5p, miR-146b-5p, miR-221-3p, miR-24-3p, and miR-26a-5p. Additionally, they identified two signaling pathways targeted by escitalopram, namely Wnt and MAPK, which are involved in MDD through cell proliferation and hippocampal plasticity [55].

### 3.3. Serotonin and Noradrenaline Reuptake Inhibitor Antidepressants

#### 3.3.1. Venlafaxine

The study by Grosse et al. showed that venlafaxine normalized the number of Tregs after seven weeks of treatment, similarly to imipramine [39,40]. A study in older adults with moderately severe MDD treated with venlafaxine for 12 weeks showed increased expression of miR-135a-5p [56].

An animal study conducted by Guan et al. showed that venlafaxine decreased the hippocampal levels of miR-204-5p and increased the expression of BDNF in chronic social defeat stress (CSDS) mice [57].

#### 3.3.2. Duloxetine

In a study conducted by Pan and Liu in rats exposed to the CUMS, a 3-week treatment regimen with duloxetine managed to restore miRNA dysregulations in the hippocampus, but not in the frontal lobe, thus indicating a possible brain region selectivity in antidepressant activity [5,58].

Lopez et al. observed a differential expression of miR-146a-5p, miR-146b-5p, miR-425-3p, and miR-24-3p depending on treatment response to an 8-week treatment regimen with duloxetine. Similarly to the study conducted by Enatescu et al. on citalopram, duloxetine was also capable of regulating the genes involved in the Wnt and MAPK signaling systems by modulating the expression of the same miRNAs [55,59].

Fiori et al. conducted a study involving two cohorts of MDD patients that received 8 weeks of escitalopram (cohort 1), desvenlafaxine (cohort 1), or duloxetine (cohort 2) treatment. In both cohorts, the baseline levels of miR-1202 were reduced, but regulated following the antidepressant treatment [60].

### 3.4. Mirtazapine

According to a study conducted by Kato et al., no miRNAs correlated with a 4-week mirtazapine monotherapy treatment response, compared to SSRIs (sertraline or paroxetine) in which miR-483.5p correlated with the improvement of depressive symptoms after 2 weeks of treatment. However, the authors suggested that mirtazapine monotherapy or combinations may be used in MDD patients who are predicted by miRNAs to not have an early response to SSRIs [61].

### 3.5. Electroconvulsive Therapy

McGrory et al. aimed to determine the normalization of E2F1 miRNA levels in peripheral blood following ECT, as this miRNA is reduced in MDD patients. However, they did not observe any change in the E2F1 miRNA peripheral blood levels after ECT and could not establish a relationship between miRNA baseline levels and depression severity or treatment response [62]. A different study conducted by Israel-Elgali et al. concluded that the PBMC expression levels of miR-24-3p and protein-coding genes FKBP5 and ITG2AB could be considered tentative biomarkers for ECT response and should be further explored [63].

## 4. Adequate Sampling for miRNAs

While consistent evidence exists regarding the potential use of miRNAs as predictors of the efficacy of antidepressant treatment, sampling represents an important issue in establishing a novel biomarker with clinical significance.

To propose a biomarker, it is important to observe alterations in accessible tissues (e.g., cerebrospinal fluid—CSF, PBMC, plasma, serum, whole blood) in different pathologies. The systematic review by Goossens et al. proposed the use of PBMC in monitoring the efficacy of antidepressants due to three of their characteristics: the ability to reflect treatment effects, the possibility to differentiate between responders and non-responders, and the baseline predictability of treatment outcomes for a broad range of interventions, such as psychotherapy or antidepressants. On the other hand, CSF is deemed inadequate due to difficulty accessing it compared to other tissues [40].

Another issue that limits the choice of sampled tissue is the location of the genes targeted by miRNAs. For example, whole blood and PBMC miRNA expression involve coding genes related to inflammation and cell viability, which have been linked to a potential epigenetic mechanism of MDD [16,40].

One advantage of miRNAs is that they could be used as multimarker models for making an accurate diagnosis, guiding treatment decisions, and assessing responsiveness to treatment [64]. Although it was demonstrated that circulating miRNA profiles are very similar in venous and arterial plasma in healthy individuals, studies on chronic inflammation and cancer detection found that overexpressed miRNA profiles were higher in arterial compared to venous plasma. Moreover, miRNA profiles in arterial plasma correlated with miRNA profiles in tissues. Consequently, blood sampling methods in human studies should be carefully chosen to determine the specific miRNA biomarkers [65,66,67].

MiRNAs are detected using different molecular methods including real-time PCR based on the PROMER technology, miRNA microarray, Northern blot analysis, in situ hybridization, and next-generation sequencing (NGS) [64,68]. Furthermore, technical challenges due to contamination by hemolysis or platelet activation, storage of biofluids, including blood, CSF, urine, saliva, and lacrimal fluid, and manipulation during the analysis process, as well as the stage of the disease could change miRNA phenotypes and expression.

Data reproducibility can be improved by controlling and optimizing experiments to reveal statistical differences in the level of miRNA expression in a certain tissue or body fluid type between study groups and healthy individuals. The accuracy of miRNA detection assays can be enhanced by progress in machine-learning techniques and bioinformatics, which facilitate better integration of multi-omics data [69].

To overcome the abovementioned challenges, the following must be carefully considered: sample size, standardized protocols at pre- and post-analytical stages, confirmation of the results of observational studies through prospective studies, as well as validation through epidemiological and functional studies [64].

## 5. Discussion

The literature data included in this minireview suggest that several miRNA expressions altered in MDD are normalized by antidepressants, indicating their potential use as biomarkers. Table 1 summarizes the relationships between antidepressant classes and miRNAs, alongside the mechanisms of modulated miRNAs in MDD without antidepressant treatment.

**Figure 1:**
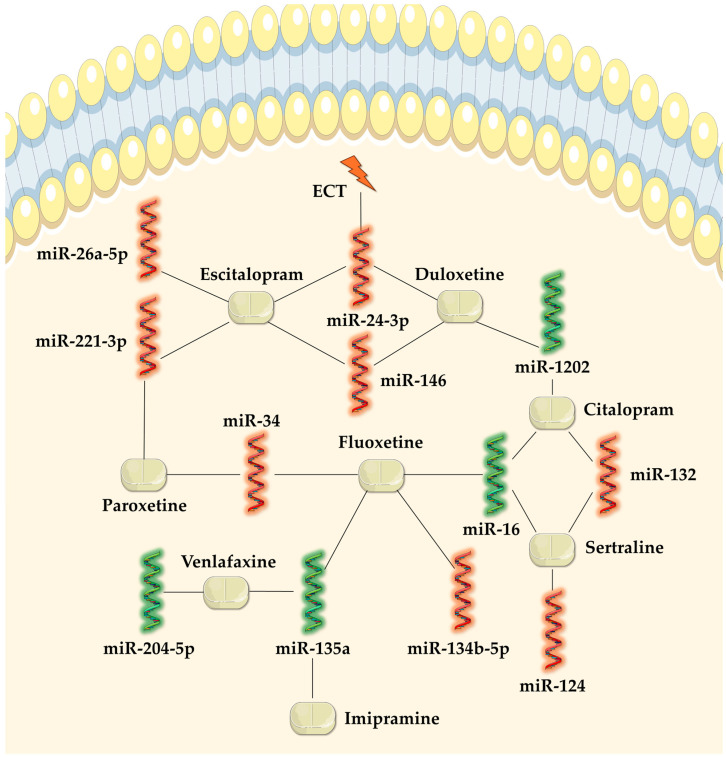
Illustrative network of relationships between antidepressants and miRNA expression. Green helixes represent upregulated miRNA expression, red helixes represent downregulated miRNA expression, tablets represent antidepressants, and a lightning bolt represents ECT.

As observed, some antidepressants identically modulated the expression of the same miRNAs. Thereby, miR-135a was upregulated by imipramine, fluoxetine, and venlafaxine, miR-16 was upregulated by fluoxetine, sertraline, and citalopram, miR-132 was downregulated by sertraline and citalopram, miR-34 was downregulated by both fluoxetine and paroxetine, miR-1202 was upregulated by both citalopram and duloxetine, miR-146a-5p and miR-146b-5p were downregulated by both escitalopram and duloxetine, miR-221-3p was downregulated by both paroxetine and escitalopram, and miR-24-3p was downregulated by escitalopram, duloxetine, and ECT. An overview of the relationships observed between antidepressants and miRNAs is illustrated in Figure 1.

The fact that several antidepressants share similarly modulated pathways supports the existence of a common epigenetic mechanism of antidepressants [72,73,74]. Therefore, the epigenetic differences between individuals may explain why the same antidepressant may have different outcomes (e.g., therapeutic success or failure) in different patients despite being used in the same conditions (e.g., adherence to adequate treatment).

Given the significant impact of inappropriate expression of miRNAs on the etiology and pathology of MDD, as well as the impaired treatment response to antidepressants, it is important to plan strategies that can mitigate inadequate miRNA responses. This requires control of environmental factors, such as oxidative stress and chronic inflammation, which can induce epigenetic modifications in gene expression through DNA methylation and changes in histone acetylation, ultimately affecting miRNAs. Therefore, another option to avoid aberrant miRNA expression is to target the enzymes involved in histone modification, namely Setdb1, G9a, PRMT1, JMJD3, HDAC2, and HDAC3. Several histone deacetylase inhibitors (HDACi) such as trichostatin, vorinostat, valproic acid, MS-275, and sodium butyrate have been employed in animal models to explore MDD treatment options [75,76,77]. As mentioned above, newer methods of mitigation include miRNA replacement therapy.

Regarding the translatability of animal studies to humans, out of all the studies on treatment response in MDD included in our analysis, only five were conducted in animals while one was conducted in both animals and humans. The best example of translatability is provided by Issler et al., who demonstrated the same modulation of miR-135a during antidepressant treatment in both animals and humans [22]. The observations on miR-34a and miR-16 according to the animal studies conducted by Baudry et al. and Lo Iacono et al. were applied to the human studies conducted by Ahmadimanesh et al. and Kuang et al. [44,48,49]. On the other hand, the impact of miR-204-3p was demonstrated only in animals [57,78,79].

Nevertheless, the clinical and genetic heterogeneity of MDD, including sex, age of onset, other psychiatric comorbidities, episode frequency, episode duration, or duration of remission, should be considered when assessing the results of human studies. Sex is regarded as one of the most important factors contributing to the heterogeneity of MDD, with women being twice as likely to develop this disorder compared to men [80,81]. Out of all the human studies on MDD treatment response included in our analysis, only two had equal proportions of male and female subjects [53,61]. Most studies had higher proportions of female participants [41,48,49,50,51,52,54,55,59,62,63], while two studies did not provide information on the number of males and females [22,60].

This minireview has some limitations, the most significant being that the literature search was conducted in a single database (Google Scholar) due to time constraints. Therefore, several important articles may have been omitted. Additionally, for better readability, we did not include details such as study design and protocol. We also failed to find studies on other antidepressants, such as tianeptine, trazodone, vilazodone, vortioxetine, bupropion, esketamine, etc.

## 6. Conclusions

The lack of a robust method to monitor and quantify the efficacy of antidepressants represents a drawback to establishing rapid and effective treatment in MDD patients. Otherwise, the lack of treatment response may cause individuals to be considered treatment-resistant when they are often pseudo-resistant.

This minireview aimed to synthesize some of the available data regarding the potential use of miRNAs as biomarkers for tracking the efficacy of antidepressants. Our analysis revealed that several SSRI and SNRI antidepressants identically modulated the expression of the same miRNAs, thus suggesting the existence of a common epigenetic mechanism of antidepressants. Additionally, we attempted to focus on some common issues associated with miRNA measurements and research in MDD, including the translatability of results from animal to human studies, the clinical and genetic heterogeneity of MDD, and the challenges of miRNA sample collection.

It is important to acknowledge the preliminary nature of our correlations and the need for further research to validate their clinical utility. Research challenges include the lack of a standardized protocol for miRNA measurement, difficulty choosing the adequate sample type and number of subjects, sample processing issues, high cost of assays, heterogeneity of the selected subjects (sex, age of onset, frequency of episodes, etc.), and ethical concerns in both animal and human studies.

In conclusion, further research to establish an effective biomarker should focus on addressing some of the abovementioned issues while improving data reproducibility of miRNA measurements.

## Figures and Tables

**Table 1:** Summary of the relationships between antidepressant classes and miRNAs, alongside the mechanisms of modulated miRNAs in MDD without antidepressant treatment.

Antidepressant Class	Relation to miRNAs	Mechanisms of Modulated miRNAs in MDD Without Antidepressant Treatment	References	
**TCA**	↑ miR-135a	Downregulated in MDD, resulting in a decreased expression of the SERT and 5HT-1A receptor in the raphe nucleus and contributing to reduced serotonergic transmission. Overexpression also maintained the depressive-like phenotype.	[22,41]	
**SSRI**				
	↓ miR-34a	Upregulated in MDD, resulting in a decreased expression of the 5HT2C receptor in the dorsal raphe nucleus.	[45]	
	↓ miR-135b-5p	Upregulated in MDD, leading to a dysfunctional expression of the SIRT1 gene, which maintains a depressive-like behavior.	[46]	
	↑ miR-16	Downregulated in MDD, leading to a decreased SERT expression and impaired serotonergic transmission.	[44,48]	
	↑ miR-451a	Downregulated expression in a CRS mouse model. Its reduced expression may affect dendritic spine plasticity through poor inhibition of the CRS-induced corticotropin-releasing factor receptor 1 expression.	[49,70]	
	↓ miR-155	Upregulated in MDD, leading to a dysfunctional expression of the SIRT1 gene, which maintains a depressive-like behavior.	[52]	
	↓ miR-124	Upregulated in MDD, reducing the expression of the BDNF gene.	[48]	
	↓ miR-132		[48,51]	
	↓ miR-221-3p	Upregulated expression in MDD was correlated with a decreased expression of SOCS1 (cytokine inhibitory signaling protein), which led to increased cellular inflammation.	[49,55,71]	
	↓ miR-146	Upregulated in MDD and involved in several signaling pathways (Wnt, cancer, endocytosis, axon guidance, and MAPK).	[55,59]	
	↓ miR-24-3p		[55,59,63]	
	↓ miR-26-5p		[55]	
**SNRI**	↑ miR-1202	Affected the expression of metabotropic receptor *GRM4*, thus regulating the glutamatergic system. Decreased peripheral levels reduced the antidepressant response and maintained depressive symptoms.	[50,60]	
	↓ miR-204-5p	Upregulated expression was associated with reduced BDNF gene expression.	[57]	
	↓ miR-425-3p	Upregulated in MDD and involved in the MAPK and Wnt signaling pathways.	[59]	
	↑ miR-135a	Downregulated in MDD, resulting in a decreased expression of the SERT and 5HT-1A receptor in the raphe nucleus and contributing to reduced serotonergic transmission. Overexpression also maintained the depressive-like phenotype.	[22,41]	
	↓ miR-146	Upregulated in MDD and involved in several signaling pathways (Wnt, cancer, endocytosis, axon guidance, and MAPK).	[55,59]	
	↓ miR-24-3p		[55,59,63]	
**ECT**				

Upwards arrows represent upregulated miRNAs and downwards arrows represent downregulated miRNAs.

## Data Availability

The data presented in this study are available in this article.
